# Community knowledge, attitude, practices and beliefs associated with persistence of malaria transmission in North-western and Southern regions of Tanzania

**DOI:** 10.1186/s12936-023-04738-5

**Published:** 2023-10-10

**Authors:** Edwin A. Liheluka, Isolide S. Massawe, Mercy G. Chiduo, Celine I. Mandara, Frank Chacky, Leah Ndekuka, Filbert F. Temba, Bruno P. Mmbando, Misago D. Seth, Daniel P. Challe, Williams H. Makunde, Athanas D. Mhina, Vito Baraka, Method D. Segeja, Yahya A. Derua, Bernard M. Batengana, Paul M. Hayuma, Rashid A. Madebe, Masunga C. Malimi, Renata Mandike, Sigsbert Mkude, Fabrizio Molteni, Ritha Njau, Ally Mohamed, Susan F. Rumisha, Deus S. Ishengoma

**Affiliations:** 1 National Institute for Medical Research, Tanga, Tanzania; 2 National Institute for Medical Research, Dar es Salaam, Tanzania; 3 National Malaria Control Programme, Dodoma, Tanzania; 4 National Institute for Medical Research, Amani Medical Research Centre, Tanga, Tanzania; 5 Swiss TPH Netcell Project, Dar es Salaam, Tanzania; 6 World Health Organization Country Office, Dar es Salaam, Tanzania; 7 Malaria Atlas Project, Geospatial Health and Development, Telethon Kids Institute, Perth, WA Australia; 8 Faculty of Pharmaceutical Sciences, Monash University, Melbourne, Australia; 9 Harvard T.H Chan School of Public Health, Harvard University, Boston, MA USA

**Keywords:** Malaria knowledge, attitude, practices and beliefs, Insecticide-treated nets, Malaria, Tanzania

## Abstract

**Background:**

Despite significant decline in the past two decades, malaria is still a major public health concern in Tanzania; with over 93% of the population still at risk. Community knowledge, attitudes and practices (KAP), and beliefs are key in enhancing uptake and utilization of malaria control interventions, but there is a lack of information on their contribution to effective control of the disease. This study was undertaken to determine KAP and beliefs of community members and service providers on malaria, and how they might be associated with increased risk and persistence of the disease burden in North-western and Southern regions of Tanzania.

**Methods:**

This was an exploratory study that used qualitative methods including 16 in-depth interviews (IDI) and 32 focus group discussions (FGDs) to collect data from health service providers and community members, respectively. The study was conducted from September to October 2017 and covered 16 villages within eight districts from four regions of mainland Tanzania (Geita, Kigoma, Mtwara and Ruvuma) with persistently high malaria transmission for more than two decades.

**Results:**

Most of the participants had good knowledge of malaria and how it is transmitted but some FGD participants did not know the actual cause of malaria, and thought that it is caused by bathing and drinking un-boiled water, or consuming contaminated food that has malaria parasites without warming it. Reported barriers to malaria prevention and control (by FGD and IDI participants) included shortage of qualified health workers, inefficient health financing, low care-seeking behaviour, consulting traditional healers, use of local herbs to treat malaria, poverty, increased breeding sites by socio-economic activities and misconceptions related to the use of bed nets and indoor residual spraying (IRS). Among the misconceptions, some participants believed that bed nets provided for free by the government came with bedbugs while others reported that free bed nets caused impotence among men.

**Conclusion:**

Despite good knowledge of malaria, several risk factors, such as socio-economic and behavioural issues, and misconceptions related to the use of bed nets and IRS were reported. Other key factors included unavailability or limited access to health services, poor health financing and economic activities that potentially contributed to persistence of malaria burden in these regions. Relevant policies and targeted malaria interventions, focusing on understanding socio-cultural factors, should be implemented to reduce and finally eliminate the disease in the study regions and others with persistent transmission.

## Background

Malaria remains a major public health problem globally and in Tanzania, and is a leading cause of morbidity and mortality, especially in pregnant women and children under the age of five years. According to recent reports, malaria was responsible for over 247 million cases and 619, 000 deaths in 2021, despite a dramatic decline which occurred between 2000 and 2015 in most of the malaria endemic countries, including some in sub-Saharan Africa (SSA) [1]. Globally, malaria incidence rate decreased by 21% between 2010 and 2015, while the prevalence decreased from 17 to 13% during the same period [2]. Tanzania, like many other countries has experienced a decline in malaria transmission in recent years [3, 4], but over 93% of the population is still at risk of malaria transmission [5]. Currently, malaria is highly heterogeneous with some parts of mainland Tanzania, like the northern and central regions recording the lowest prevalence as low as 5.1% and 2.8%, respectively, while the southern, western and north-western regions have the highest burden, with parasite prevalence between 33 and 38%, respectively [6]. The reasons and factors associated with persistence of malaria in some regions of Tanzania are not clearly known.

Effective control of malaria in Tanzania is based on an integrated approach that targets the vectors, parasites, and human host. The main interventions currently used by the National Malaria Control Programme (NMCP) for vector control include long-lasting insecticidal nets (LLINs), indoor residual spraying (IRS) and larviciding [7]. Case management interventions include, prompt diagnosis using rapid diagnostic tests (RDTs) and effective treatment using artemisinin-based combination therapy (ACT). The main chemo-preventive measures include intermittent presumptive treatment in pregnant women (IPTp) using sulfadoxine/pyrimethamine (SP) [8–11]. With respect to human hosts, the Tanzanian NMCP implements social behaviour change and communication (BCC) interventions to (i) reinforce and update malaria knowledge among community members and appropriate prevention, testing and treatment methods; (ii) increase knowledge amongst vulnerable populations/groups with high risk of malaria, and prevention and treatment options available to them; and (iii) influence social norms about healthy behaviours around malaria prevention and care [12]. In a big country with high geographical and population diversity, implementation of malaria interventions is not often targeted considering the social-cultural variations at macro and micro geographic levels.

For the fight against malaria to be successful, all approaches must be carefully coordinated. For example, the government and private partners must ensure all intervention options are put in place, while the community must be aware of and ensure uptake, and appropriate use of the interventions. The degree by which the communities comply to this requirement highly depends on their level of knowledge (understanding, perception and interpretation), attitude and practice (KAP), and beliefs towards malaria, its prevention and control [13]. Efforts to fight malaria may be slowed down if the KAP and beliefs of the target communities are not favourable or considered in designing and implementing different interventions. Studies conducted in Tanzania and elsewhere on KAP and beliefs found that most community members possessed reasonable knowledge of malaria, its transmission, symptoms, and prevention measures, as well as favourable attitude and practices such as sleeping under bed nets, timely seeking of services from appropriate sources (particularly health facilities) and rational use of ACT for malaria treatment [13–17]. However, some individuals within the communities had limited knowledge of malaria, modes of transmission, and undesirable practices for effective treatment of malaria, and its prevention and control, such as delayed healthcare-seeking behaviour, seeking treatment from informal sectors, self-medication with a variety of local herbs, and consulting traditional healers [16, 18–21].

Different studies on KAP and beliefs have shown that the impact of malaria interventions can be negatively affected by poor practices among community members [22]. A study conducted in Ethiopia reported that some people considered exposure to cold, drinking dirty water, and hunger as important causes of malaria [23]. In some communities, there are other practices, knowledge issues and health care system issues such as concerns about side effects of anti-malarial medications and insecticides, and shortage of healthcare facilities and health workers that significantly affect access to, uptake, utilization and effectiveness of malaria interventions [24]. Research on KAP and beliefs is paramount to achieving intended malaria control outcomes and to enhance the knowledge about the contribution of these factors to the recently observed changes in malaria burden including persistence of transmission in some areas. This study covered regions from North-western and southern Tanzania which have consistently reported high malaria burden over the past 20 years and utilized socio-anthropological qualitative methods to determine KAP and beliefs of community members on malaria. It also assessed if these and other related factors might be associated with increased risk of and persistence of malaria in North-western and southern regions of Tanzania.

## Methods

### Study design and area

This was part of a bigger cross-sectional study which was conducted between September and October 2017 and covered 16 villages in eight districts (two villages per district) from four regions of Geita, Kigoma, Mtwara and Ruvuma as previously described [25]. The main study aimed at broadly assessing the factors associated with persistence of malaria in the selected study regions in order to generate evidence for reducing the burden of the diseases and supporting the ongoing elimination efforts in Tanzania. This component focused on KAP and belief of community members and service providers as potential factors contributing to the observed epidemiology of malaria in north-western and southern Tanzania over the past 20 years. Qualitative methods were employed with the aim of exploring KAP and beliefs of community members and service providers about malaria, its control and prevention. These Regions have persistently high malaria transmission as shown in different surveys conducted in Tanzania between 2011 and 2017. Such surveys include the 2011–2012 Tanzania HIV and Malaria indicator survey (THMIS), 2015–2016 Tanzania demographic and health survey (TDHS) and the 2014/2015, 2017 school malaria parasitological surveys (SMPS) [26].

**Fig. 1:**
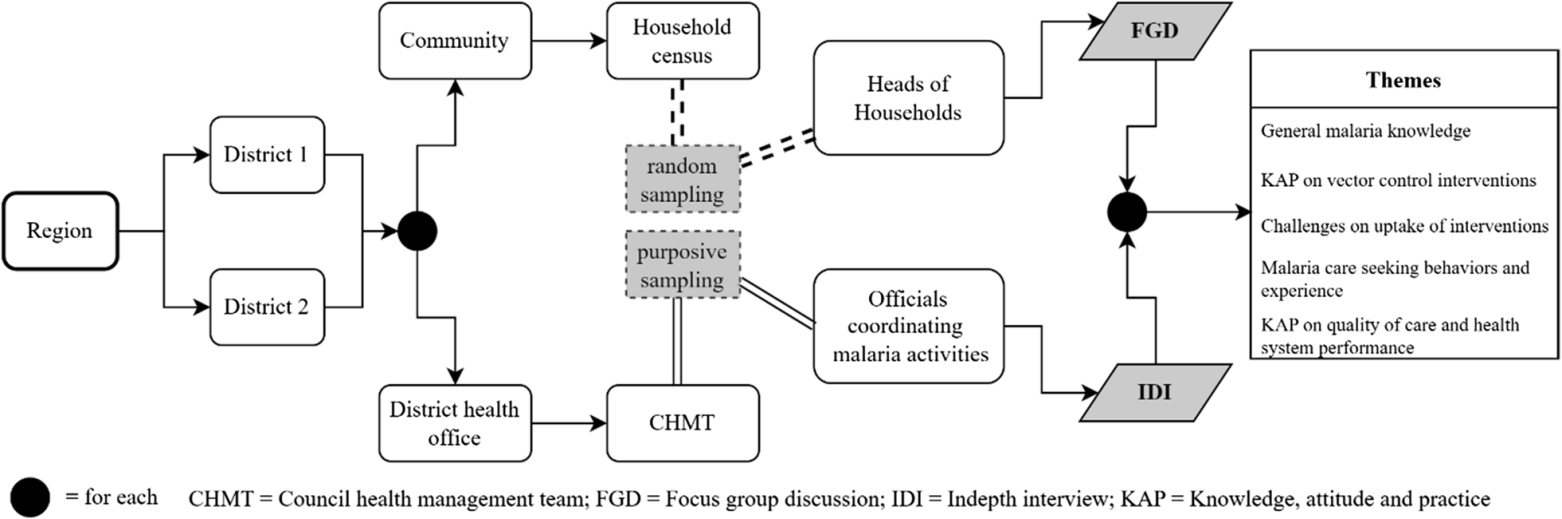
Flow chart showing the study design and workflow

The design and workflow of this study are illustrated in (Fig. 1), whereby community members from the study villages and service providers at the district level were targeted. Two districts were purposively selected from each of the four regions; Buhigwe and Uvinza (Kigoma), Mtwara DC and Nanyumbu (Mtwara), Tunduru and Nyasa (Ruvuma) and Nyang’hwale and Chato (Geita). The two districts in Geita region were chosen with an added criterion of taking part in IRS activities which were done shortly before this study. In the target districts, two villages with high malaria burden, based on the number of cases reported by health facilities in 2016, were purposively selected, making a total of 16 villages. The selected villages were located within the catchment area of one of the health facilities which were selected for the main study and they were reachable by road from the district headquarters. The villages were also located in different parts of the regions to capture wider geographic coverage of the regions. Two Focus Group Discussions (FGDs) from each village, included both males and females heads of household (HHs) were randomly selected from the sampling frame of HHs heads who did not participate in the parasitological survey and KAP interviews using computer-generated random number as described by Chiduo et al. [25]. Purposive sampling technique was used to select 16 key informants for in-depth interviews (IDIs) which were undertaken with at least two officials (District Medical Officers—DMOs and District Malaria Focal Persons—DMFPs) involved in malaria control at the district level.

### Study population, inclusion and exclusion criteria

The target population included male and female heads of HHs who were involved in FGDs and DMOs and DMFPs from the eight districts (Uvinza, Buhigwe, Chato, Nyang’hwale, Nyasa, Tunduru, Nanyumbu, and Mtwara) who took part in IDIs. FGDs targeted the heads of HHs (both male and female) who were randomly selected from each of the study villages, with the ability to communicate fluently in Kiswahili and were aged ≥ 18 years old. All participants were asked to provide consent to take part in the study. Exclusion criteria included absence from the study areas at the time of data collection, not giving informed consent and illness or any other condition which made the participant unable to be interviewed.

### Data collection procedures

Interview guides with open-ended questions in Kiswahili, the most widely spoken language in the country, were developed and used to facilitate data collection. Research tools (Interview guides) were prepared and tested among volunteers who were sampled around Tanga City prior to the actual data collection. The purpose of the pilot study was to evaluate the suitability of the tools, the flow of topics and questions, participants’ comprehension of the questions, and the ability of researchers and their assistants to properly conduct the interviews. During the pilot study, emphasis was also placed on following ethical principles of informed consent, voluntary involvement, and maintaining confidentiality. Before the actual field work, the study tools were modified and updated to cover the changes which were deemed necessary during the pilot testing.

A total of 32 FGDs with male and female participants and 16 IDIs with DMOs and DMFPs were conducted. Each FGD comprised of 8–12 participants, with male and female participants in separate groups. The venues for the FGDs and IDIs were organized with assistance of local village leaders in each village. Prior to the discussion, study objectives and other relevant study information were explained to the participants. The FGDs and IDIs were conducted by experienced moderators and FGDs were held in a ‘round table’ manner. Personal information about each participant (e.g., age, sex, education, and occupation) was collected before the discussions. Each participant was given a unique identification number (ID) chronologically, and personal information was recorded using a checklist designed for FGDs and IDIs. Each FGD took 45–60 min and IDIs took 30–45 min, and were conducted in a setting convenient to the participants. Interviews were audio-recorded in addition to taking notes with permission from each participant.

Data collected in the FGDs and IDIs included general knowledge of malaria (its transmission, signs, symptoms and treatment), perceptions and attitude towards malaria control interventions, barriers to uptake of different control interventions, health care seeking behaviour and perceptions of quality of care and health system performance.

The goal of conducting 32 FGDs across four regions was to gather different viewpoints, experiences and first-hand accounts from various contexts within the specific setting. Sixteen IDIs were conducted with DMOs and/or DMFPs to gather their perspectives of malaria. DMOs and DMFPs were the key informants for this study since they were the primary officers in charge of overseeing all matters pertaining to malaria and its control in their respective districts. FGDs and IDIs were held continuously until the saturation threshold was achieved.

### Data processing and analysis

Interviews were conducted in Kiswahili to reduce the impact of language and cultural barriers. All interviews were audio recorded with permission from participant(s) and transcribed verbatim by the research team, and later translated into English for analysis. Data analysis was done manually using thematic analysis framework [27]. Classification and analysis of the data was independently done by two moderators to enhance the credibility of the different categories that emanated from the interviews. To deepen insights and ensure the findings addressed the research questions and reflected the research context, analysis of data was regularly reviewed by members of the research team who carried out data collection. A coding framework was initially developed by one researcher based on the predefined objectives of the study and further refined after preliminary review of the data to understand the narratives provided by participants. Inductive coding was used to identify sub-themes within each of the key themes. The study objectives and research questions informed the decision of which participant narratives to use. The narratives that accurately and fully addressed the research objectives were given the highest weight, regardless of how many or how few individuals cited them.

## Results

### Demographic characteristics of study participants

**Fig. 2:**
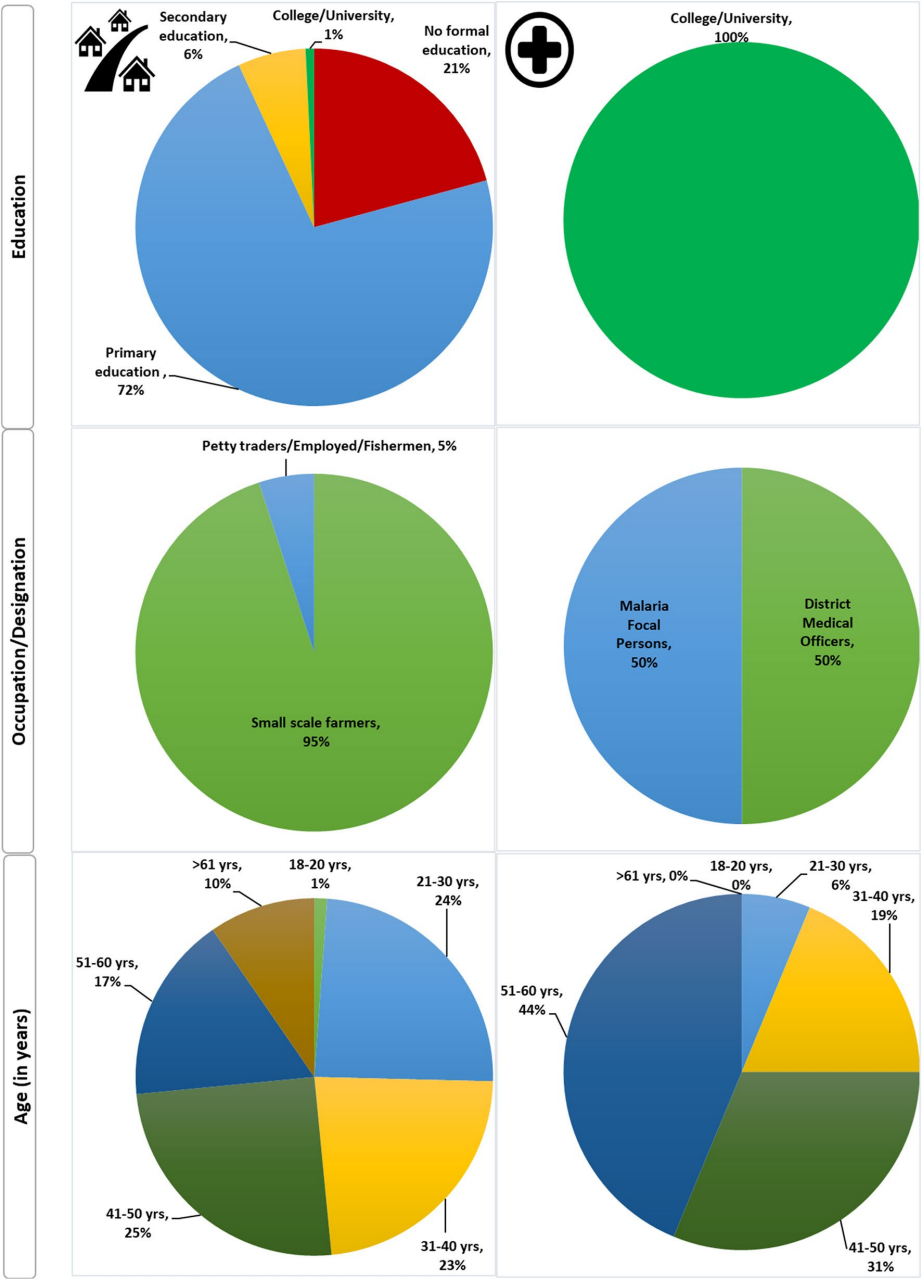
Socio-demographic profiles of FGD (left panel) and IDI (right panel) participants

A total of 276 individuals participated in the present study whereby 260 took part in the FGDs while 16 took part in IDIs. FGD respondents’ age ranged from 18 to 85 years. Most (~ 75%) of HHs were below 50 years while most of IDIs participants (75%) were between 41 and 60 years old, with the remaining quarter aged 21–40 years (Fig. 2). A high proportion of FGD participants (72.3%) had primary education; and there was a significant difference in the level of education attained by participants of different sex; with more male participants who had higher levels of education than female. The main occupation of FGD participants was small-scale farming (95.0%). The IDIs participants (mainly DMOs and DMFPs) had College or University education and their age ranged from 27 to 59 years and 12 (75.0%) were males (Fig. 2).

### Key themes which emerged from FGDs and IDIs

The findings of this study are presented within the following five themes, which emerged during the analysis of the FGDs’ and IDIs’ transcripts: the themes include: (1) general knowledge of malaria (transmission and causes; and prevention); (2) reasons for low use of mosquito vector control interventions, long-lasting insecticidal nets (LLINs) and indoor residual spraying (IRS), such as LLINs/IRS & bedbugs; LLINs and erectile dysfunction; LLINs usage and climatic conditions; LLINs & poverty; and misconceptions on IRS); (3) IRS and larviciding use and related challenges; (4) malaria health-seeking behaviour and care experiences (self-medication, decision to visit the health facility, and the use of local herbs); and (5) availability of health workers and health financing [use of and dissatisfaction with the community health fund (CHF) services].

In the next sections, the term ‘bed nets’ has been used in quotes in place of LLINs unless stated otherwise, similarly the term ‘participants’ refers to either FGDs’ or IDIs’ respondent unless stated explicitly.

### General knowledge of malaria (cause, transmission, symptoms and prevention)

#### Cause and transmission

FGD participants were asked about the cause of malaria, its symptoms, transmission, and how it can be prevented. The majority were aware of its symptoms and that mosquitoes transmit malaria. In general, they understood that the disease is transmitted by a mosquito, and in many cases, most of the participants mentioned that only female mosquitoes are responsible for transmitting malaria. This was well articulated as referenced by this 29-year-old participant:

*“Malaria is transmitted by a female mosquito called Anopheles” (P 127-FGD-Female-29yrs- Geita)*

Another participant echoed what was spoken by others:

*“Malaria is a disease transmitted by mosquitoes. Not all mosquitoes can transmit malaria but female mosquitoes called Anopheles are the ones responsible for transmission of malaria” (P 15-FGD-Female- 30yrs- Kigoma)*

However, not all FGD participants possessed the correct knowledge of malaria and its transmission. A misconception about malaria transmission was that the disease could affect someone through different routes apart from a bite from an infected mosquito. Some of the participants were of the view that malaria parasites get into the human body through bathing and drinking un-boiled water, or consuming contaminated food that had malaria parasites without warming it. For example, some participants had this to say:

*“Here in our village, the majority of us are using water from the river for bathing and drinking. Water from the river is not safe, and we are drinking this water without boiling; that is why we have malaria in our community.” (P171-FGD-Male-24yrs-Ruvuma)*

Another participant explained that:

*“I urge everyone to stop eating contaminated food, especially leftovers. The majority of the people here do not properly cover their leftovers, and when they wake up in the morning, they quickly rush to eat such food without heating it. During the night, mosquitoes tend to leave parasites on the food, and when someone eats leftovers, they will get malaria” (P 172- FGD- male-27yrs- Ruvuma)*

What was interesting in these two perceptions about water and food in relation to malaria transmission was the association of heating or boiling as a preventive measure. While FGD participants could debate about transmission; the actual cause of malaria which is the parasites, was not well articulated. It was narrated by some participants that in the community, some people still think that demonic possessions are to blame for and are believed to be a cause of complicated/severe malaria which sometimes lead to convulsions.

For example, an IDI participant said:

*“Public awareness regarding complicated/severe malaria is still low. There are different beliefs among community members; for example, if a child gets severe malaria with convulsions, they say the child was possessed by demons and parents do not take the child to the health facility, instead they send him/her to a traditional healer. Eventually, the child ends up dying there” (P 271-IDI- 53yrs- Ruvuma)*

#### Prevention

Strategies of malaria prevention were well articulated in all FGDs and IDIs. Participants reported preventative methods categorizing them into those used inside the house and those applied outdoors. Most of discussants reported to rely on LLINs to protect themselves from mosquito bites. They reported that they have been hearing about LLINs in different media outlets for many years. Nearly all participants reported LLINs as the main malaria prevention method:

*“The way to protect yourself, personally I think we should have bed nets. Because if you don’t use a bed net malaria can hit you” (P 222-FGD-Male-54yrs-Mtwara)*

*“We protect ourselves using bed nets when we go to sleep” (P 89-FGD-Female-32yrs-Geita)*

An IDI participant also supported what were reported by FGD participants:

*“Most of the people in the community prefer to use bed nets; if you educate them properly they make good use of nets” (P 263-IDI- 51yrs- Kigoma)*

Participants were aware of the strategies used to prevent mosquitoes outside their houses including destroying standing water bodies which provide breeding sites, trimming grasses and shrubs in peri-domestic areas and use of local herbs such as burning eucalyptus leaves to expel mosquitoes. However, participants from each of the study regions possessed their own types of herbs which they used to expel mosquitoes. For example, a participant from Geita reported use of eucalyptus while those from Ruvuma reported to use other types of smelly leaves:

*“For example, there are leaves called “MNUNGANGU” (a YAO word which means a leaf with very bad smell) which are normally used to deter mosquito” (P 271-IDI-53 yrs-Ruvuma)”*

*“In the past, we were using eucalyptus leaves to deter mosquitoes. When you burn the green leaves, mosquitoes will disappear. But now we are using bed nets” (P 129-FGD-Female-29 yrs-Geita)*

A participant echoing his fellow discussant said:

*“We normally protect ourselves by using “MALUMBA” (a Sukuma word) leaves; once you put them in the house, they release smell and mosquitoes vanish from the house” (P 98-FGD-Male-37 yrs-Geita)*

IRS and larviciding were mentioned by both FGD and IDI participants as important tools in the prevention of malaria outdoors. The emphasis and importance of IRS and larvicides were mainly reported in Geita Region that had extensive experience of using them. However, even in the regions such as Mtwara and Ruvuma where IRS was yet to be implemented, both FGD and IDI participants acknowledged its potential. They associated persistence of malaria in their regions with a possible lack of these important control tools (IRS and larvicides):

*“We are being asked by the people in the community, why are you always pushing people to use bed nets only as a means of fighting malaria? Why don’t you think of another way to fight malaria such as IRS? Even me as a malaria focal person, I agree that we need to employ this strategy of IRS. We have never sprayed chemicals in this District” (P 271-IDI-Male-53yrs-Ruvuma)*

An FGD participant added;

*“Perhaps what can be done to prevent malaria is that; we get chemicals which can be sprayed in all mosquito breeding sites in order to destroy the mosquito eggs and kill the mosquito themselves completely so that there is no further reproduction of mosquitoes” (P134-FGD-Male-36yrs-Ruvuma)*

### Reasons for low use of vector control interventions (LLINs and IRS)

Despite the fact that participants acknowledged the use of LLINs for malaria control, there were different views that suggested that LLINs were infrequently used and sometimes misused. Although most participants reported that they possessed LLINs, their use was mostly hindered by factors related to LLINs quality and misconceptions.

#### LLINs and bedbugs

The majority of FGD participants reported that LLINs, especially those provided free by the government and distributed during the national campaigns, brought a lot of bedbugs. When asked to clarify, most participants were not aware of how bedbugs were brought by LLINs. Participants in Ruvuma region however, believed that LLINs were infested with bedbug’s eggs at the time they were distributed to community members. Because of that, some participants mentioned that they decided to abandon the LLINs. This response from one participant in Ruvuma resonates well with this popular view around bedbugs and LLINs:

*“I agree those bed nets which were given for free by the government brought bedbugs. In my house, there was no single bedbug before, but after getting the bed net, I saw many bedbugs. I decided to throw it away and buy another one from the shop; to date there is no bedbug in my house” (P170-FGD-Male-24 yrs-Ruvuma)*

In addition, when an IDI participant was asked if there are any issues related to the use of LLINs, he replied:

*“Yes. Very much! I am saying very much because we have evidence of these claims. There are some areas where people report that free nets provided by the government and distributed by district officials bring bedbugs” (P 271-IDI-Health officer—Male-53yrs-Ruvuma)*

#### LLINs and erectile dysfunction

Another misconception related to possible low LLINs use was that, it was reported to cause erectile defects and low libido in men (suggested that sex drive in men decreased by using LLINs). This was widely reported by participants in FGDs, although other participants vehemently refuted this claim. In elaborating on how actually LLINs caused low sex drive, participants associated it with the lowered ability of men (who use LLINs) to sexually satisfy their sexual partners:

*“Ooh yes! It is true when you sleep under a bed net, you cannot sexually satisfy your partner because you can ejaculate once, but if you sleep without a bed net you can ejaculate thrice” (P167-FGD-Male-44yrs-Ruvuma)*

This view was also echoed by an IDI participant:

*“To be honest, for men to use bed nets it is a challenge. There were some rumours; men believe that sleeping under the bed net makes them weak during sexual activity. I remember sometimes ago when there was a national campaign of distributing free bed nets which were donated by the Americans, those bed nets with Zebra lines, men claimed that when you sleep under those bed nets, you can clearly smell the chemical. The chemical was claimed to cause low sexual desire among men” (P269-IDI-Male-27yrs-Ruvuma)*

#### LLINs and climatic conditions

Low LLINs use was also associated with seasonal variations. Participants generally perceived abundance of mosquitoes that may cause malaria to be in the rainy seasons and immediately after and therefore it was important for them to use LLINs during these periods. In addition, it was reported that sleeping in a bed covered with a bed net *(not exclusive for LLINs)* during warm and humid weather, the room becomes hot with no air movement. The heaviness of the air was reported to somehow affect the ability of the users to breathe properly and caused discomfort, in their attempt to enjoy their sleep:

For example, a participant said:

*“There are some people who say that they do not prefer to sleep under bed nets (not exclusive for LLINs), especially in this month of September because the weather is warm; they say the bed nets increase warm condition while sleeping and they decide to remove them, while in this month of September there are mosquitoes everywhere” (P16-FGD-Female-30yrs-Kigoma)*

An IDI participant was with the view that, his clients oftentimes did not use bed nets (not necessarily LLINs) in both the warm and wet weathers:

*“During warm season, people do not prefer to use bed nets (not necessarily LLINs) because it worsens the heat. On the other hand, during the cold season in June and July mosquitoes tend to change behaviours, their wings become thicker because of the cold weather, so you cannot hear mosquito's noise. People say in cold season there are no mosquitoes as a result they do not use bed nets” (P 271-IDI-Male Health officer–53yrs-Ruvuma)*

#### LLINs and poverty

In the study regions, participants reported that most low-income families are unable to afford LLINs. Moreover, it was reported that the majority of low-income communities have poor-quality housing, which makes it difficult to use LLINs.

For example, a participant said:

*“Poverty contributes to malaria persistence because someone cannot afford to buy a bed net” (P 125-FGD-male-26yrs-Geita)*

In agreement, another participant added:

*“Poverty contributes greatly to the increase of malaria. Although we are getting free bed nets from the government, we have poor-quality houses. We also sleep on the floor where there is no proper place to hang and fix the bed nets” (P 120-FGD-Male-56yrs-Geita)*

#### Misconception regarding IRS

IRS has been used for vector control in some Regions in the Lake zone of Tanzania including Geita. FGDs participants had some concerns with IRS which are similar to those raised by the users of LLINs. They claimed that chemicals used for IRS facilitated the appearance of bedbugs in the sprayed houses. Participants believed that bedbugs appeared and increased in the houses after spraying. However, when probed further, they could not explain exactly how this was possible but to them, it was what they experienced. For example, an FGD participant reported:

*“We do not like the chemicals used for IRS because they bring bedbugs in our houses. The houses sprayed tend to have more bedbugs than those houses which have not being sprayed” (P 127-FGD-Female-29yrs-Geita)*

When an IDI participant was asked to give her opinion related to the acceptability and misconception related to the use of IRS, she had this to say:

*“The IRS program has been implemented in this District for the past 5 years. During early years of implementing IRS program, there was a very good response and acceptance from the community. However, last year the campaign encountered several challenges since some people were reluctant to cooperate. People in the community had beliefs that the chemicals used for IRS bring bedbugs in their houses while others see IRS as a disturbance because they are asked to shift their domestic items outside the house and then bring them inside again. Others claimed that the chemicals have bad smell” (P 265-IDI-Health officer-Female-32 years old-Geita)*

### IRS and larviciding use and related challenges

Participants from regions that had implemented Larviciding and IRS acknowledged the benefits of these malaria control interventions. The majority of participants, especially in the Geita region, reported that they have witnessed regular implementation of the IRS, and a few reported about larviciding. They specifically noted that in the beginning when these interventions were implemented, there was a sustained reduction of mosquitoes and malaria cases. But in their views, these positive outcomes have not been sustained and raised suspicion about the implementation of the interventions, which possibly resulted into re-emergence of mosquitoes and increased malaria cases in the communities.

Some participants reported ineffectiveness of both interventions explaining that dishonest individuals entrusted with the task of mixing and spraying, over-diluted the chemicals used for IRS. They further reported that some of these unfaithful individuals stole IRS or larviciding chemicals, an act that deeply jeopardized effective spraying and larviciding efforts. Others reported that the quantity of IRS chemicals which is allocated to them is always inadequate:

*“Those who are involved in spraying IRS are not faithful, they sell the chemicals. They mix small amounts of chemicals with water and sell the rest” (P115-FGD-Female-28yrs-Geita)*

In agreement with the above, one participant from the same region had this to say:

*“I do not like IRS chemicals, they are just over diluted and I feel that they just spray water on the walls of our houses” (P114-FGD-Female–25yrs-Geita)*

An officer from Kigoma was concerned with the quantity of the larvicides provided:

*“We have been given larvicides but are not sufficient for the whole district. We were given only 45 cans” (P265-IDI–Male-39 years old-Kigoma)*

Indeed, there were some participants who offered a way out to the above challenges. They acknowledged the potential benefits of IRS and saw that there was an opportunity to improve on the current practice. To them, it seemed that IRS as an intervention demanded more community engagement, continued education and public communication to increase its acceptance. A FGD participant from Geita had these words that were well supported by the rest of the participants in his group:

*“Chemicals used in IRS are effective; the primary issue is to educate the community so that they can understand the whole exercise of IRS.” (P 120-FGD-Male-56yrs-Geita)*

Comments from other participants alluded to household owners’ reluctance to have their houses sprayed fearing the chemicals:

*“The chemical used for IRS are really good, but sometimes the community itself is the problem. You will find some people do not want the chemicals to be sprayed inside their houses, they just tell the sprayers to only spray the chemicals outside the house. But generally, the chemicals are good” (P 118-FGD-Male-35yrs-Geita)*

### Malaria health seeking behaviour and health care experiences

#### Self-medication

Health care seeking for malaria became an important theme that all participants had something to talk about. Two main practices dominated: first, participants reported to self-medicate before reporting to health facilities for check-ups whenever they experienced malaria-related symptoms. In case symptoms persisted, that is when they decide to visit health facilities. They usually go to the drug outlets and buy pain killers such as paracetamol (Panadol) and/or even purchase anti-malarial, popularly known as “Mseto” (a Kiswahili term for “combination drug” and may be used to refer to artemether-lumefantrine—ALu or other artemisinin—based combinations—ACT). In other words, presentation at health facilities is done late when malaria has become complicated:

*“I will speak the truth, we are making mistakes. In our village we have a tendency when someone is sick, we usually rush to the drug outlet to buy drugs. We are taking medications without knowing what we are suffering from. This is a mistake” (P229-FGD-Male-64yrs-Mtwara)*

When an IDI participant was asked about health-seeking behaviour, he gave this statement:

*“No! Health care seeking behaviour is still low, extremely low, very low. For example, when a child has fever or even an adult, community members tend to hurry to the drug shop to buy Panadol and when they have temporary relief they will go to the farm. Remember this person is in the early stages of uncomplicated malaria, then after two days that child may develop complicated/severe malaria. Some of the community members do not adhere to the recommendations of seeking health care services from health facilities within 24 h after early symptoms of suspected malaria” (P271-IDI-Male-53yrs-Ruvuma)*

#### Decision to visit the health facility

Some FGD participants reported to seek treatment for suspected malaria by visiting the health facilities. These facilities were mostly government dispensaries and health centres. Moreover, some participants suggested that sometimes it really depends on someone’s views and preferences because there are several factors that influence decision making of where to seek medical care from, as explained below.

*“When I feel I have symptoms of malaria, I always rush to the dispensary” (P 250-FGD-Female-49yrs-Mtwara)*

Another participant had this to say,

*“It is true, you know for this case, everyone has his/her own understanding, others prefer to go to the dispensary, others go to the hospital directly for testing, it really depends on someone's understanding” (P 55-FGD-Male-60yrs-Kigoma)*

Another participant added:

*“We always rush to the pharmacy to buy drugs because it is cheap. The majority of us avoid going to the dispensary where there are experts because we don’t contribute to the CHF (community health fund) services” (P 26-FGD-Male-39yrs-Kigoma)*

In agreement with the above, one participant from Mtwara Region had this to say:

*“We always prefer to go to the pharmacy because when you go to the dispensary you pay a lot of money” (P 251-FGD-Female-39yrs-Mtwara)*

#### Use of local herbs

In Kigoma and Mtwara regions, some participants reported the use of local herbs to treat malaria symptoms and they got temporal relief after using such herbs. Although the practice was not widely reported, it suggests that other avenues are being employed to address malaria related challenges:

*“There are some people who still use traditional medicine to treat malaria. They use the leaves of “MLULUNGUNJA” or “MFUMYA” trees. They grind the leaves, strain them, mix with water and drink. When they have temporary relief, they ignore going to the health facility” (P09-FGD-Female-40yrs-Kigoma)*

Interestingly, this local herb can be taken orally or per rectal famously known as “KUHINIKA” in local language, using a small pipe that is inserted per rectal. To support this assertion, a FGD male participant from Kigoma, 51 years old had this to say:

*“Yes, others are taking this traditional medicine through “KUHINIKA” (A liquid local herb taken through the rectal route). In Kiswahili language it is famously known as kupiga bomba (to pump dirt out of the rectum). After a while, the patient will have very serious diarrhoea, and all dirt will come out from the stomach and malaria patient will have some relief” (P 07-FGD-Male-51yrs-Kigoma)*

In Mtwara, the use of herbs to treat malaria was done through consumption of leaves from the popular neem tree (*Azadirachta indica*), locally known as *MUAROBAINI*:

*“When someone has malaria symptoms, nowadays there is “MUAROBAINI”; they grind the leaves of MUAROBAINI, boil them and drink. When they drink the extracts, they get relief, other people boil the leaves of MUAROBAINI and inhale its vapour” (P 09-FGD-Female-35yrs-Mtwara)*

### Availability of health workers and dissatisfaction with the community health fund (CHF)

Almost all participants reported issues related to availability of health workers and implementation of CHF and how they affect access and utilization of health care services for malaria in their communities. Participants in all four regions reported that there were insufficient numbers of health workers in their areas to satisfactorily attend malaria cases. They stated that it is common to find only one health worker in a dispensary that serves a lot of people. For example, an IDI participant from Kigoma had this to say:

*“With regard to staffing level, we are understaffed by 64%. We really have shortage of staff in all carders: clinicians, nurses and laboratory staff” (P262-IDI-Male- 39yrs-Kigoma)*

In support of the above assertion, one FGD participant said:

*“I am not happy with the health care services at our dispensary. First there is a shortage of qualified health workers to the extent that even health attendants are attending patients with serious illnesses. At our dispensary, there is no clinician but only nurses and health attendants” (P 170-FGD-Male-32yrs-Ruvuma)*

In agreement with the above comment, another FGD participant said:

*“We are not satisfied at all with the number of health workers. The issue is, we have only one health worker here and above all he has his own problems too” (P 04-FGD-Male-32yrs-Kigoma)*

On the other hand, the majority of the participants, especially in FGDs, expressed their dissatisfaction with the CHF services. They lamented that CHF services are very poor, as there are always no medications, and some health workers are also very rude. Others claimed that there is no difference between CHF users and non-CHF beneficiaries. Participants reported that CHF is ineffective, and the majority of community members hesitate to join the scheme due to the poor quality of their services. For example, an FGD participant said:

*“I am a beneficiary of CHF. But one day when I was sick with suspected malaria, I went to the dispensary with my CHF card, when I arrived at the dispensary the health worker refused to attend me. He told me that, I am still young and energetic therefore I must contribute some money to be able to get treatment, claiming that CHF is only for children and the elderly” (P 259-FGD-Male-29yrs-Mtwara)*

Another participant commented:

*“We have a CHF program in our village, but this program is ineffective because our clinician is often absent from work” (P 04-FGD-Male-35yrs-Kigoma)*

Another participant added:

*“The majority are fearful to join CHF because their services are poor. Every time you go to the health facility they will tell you there are no drugs and ask you to buy drugs from the pharmacy” (P 129-FGD-Female-29yrs-Ruvuma)*

There were mixed opinions among IDIs participants regarding the services provided by the CHF scheme. Some denied but others admitted that there were some valid challenges and complaints from community members about CHF. In addition, they reported other operational and administrative challenges; for example, some participants reported that:

*“We have challenges; there are some health workers who are not faithful, they steal CHF money. There is one health worker at a certain facility who stole 1.5 million Tanzanian shillings (~ 650USD), this particular person has been suspended to date” (P262-IDI- 39yrs -Kigoma)*

Another participant said:

*“The majority do not bother joining CHF, claiming that its services are poor. People say that every time you go to a health facility, you will be told there is no medicine. This is the main reason why people hesitate to join CHF” (P 275-IDI-Female- 35yrs-Mtwara)*

Another participant had this view:

*“CHF coverage is low, the coverage is about 40%, regional wise. The eagerness to join the scheme is minimal because potential members say that there is no difference between CHF users and non-CHF beneficiaries” (P 271-IDI-Male-53yrs-Ruvuma)*

In contrast to the above statement, some IDIs participants attributed the shortage of drugs to systemic challenges that are now a thing of the past. One participant commented that:

*“In the past, CHF services were poor and were not accepted by many people because we had no drugs, but now we have sufficient drugs” (P 265-IDI-Health officer-Female, 32yrs-Geita)*

## Discussion

This study was undertaken as part of a bigger study and assessed KAP and beliefs of community members and service providers in an attempt to determine factors which might potentially be associated with increased risk and persistence of malaria transmission in high burden regions of western, north-west and southern Tanzania. The findings revealed that study participants had a good understanding and general knowledge of malaria, specifically about its cause, transmission, symptoms and prevention. The study also revealed that there were issues related to health-seeking behaviour for malaria and other myriad forces resulting in low utilization of existing vector control interventions, which, combined with socio-economic issues, might have resulted in ineffective malaria control interventions in the regions. Furthermore, pertinent issues around malaria care experiences arose as significant barriers to effective case management and patient dissatisfaction. These findings are discussed below with reference to the broader malaria control strategies that continue to be implemented in Tanzania and other endemic countries.

The findings showed that most of the community members had good knowledge of malaria, including its cause and how it is transmitted, its symptoms and how it can be prevented. The majority were aware that female *Anopheles* mosquitoes are the sole vectors responsible for the transmission of malaria parasites. The level of knowledge possessed by participants could be associated with sustained malaria control campaigns in the country and at different levels. These have ranged from advocacy about the disease in the media [28] to focused malaria orientation to pregnant women and mothers at clinics [29]. However, some cultural beliefs were reported regarding the cause of malaria and these were associated with delays to seeking care at health facilities [30] leading to self-medication and use of local herbs. These findings suggest that culturally sensitive strategies should be used while educating communities on malaria.

Prevention of malaria using LLINs was well articulated and reported by participants as the cornerstone/hallmark of malaria control. It was interesting to hear the myriad control measures for mosquito bites outside the houses. Although not a novel finding [21], the continuous reliance on local herbs to keep mosquitoes away feeds into the ongoing debates on the control of malaria (outdoor biting) given the shifting biting trends from indoor to outdoor. How effective are these local tools especially taking into consideration the context within which they are applied and how does context (high or low altitude, humid or dry) affect their potency are key issues which are not clearly known. In addition, it is not clearly known how can these approaches be harnessed and repackaged, and yet remains affordable, available and accessible to rural communities where malaria is still a problem. These and other questions need to be addressed in order to exploit local knowledge and practices in the ongoing strategies to eliminate malaria in Tanzania by 2030.

LLINs and other malaria control tools that are impregnated or infused with insecticides have been demonstrated/ documented to decimate other vectors [31] but also irritate others [32]. In relation to the latter, it has led to community reluctance to use these malaria control interventions believing that the insecticides in LLINs or those used for IRS actually bring or increase bedbugs and other insects such as cockroaches, fleas and lice [33]. As reported by study participants, the emergence of bugs may truly be caused by the insecticides in the LLINs. The problem is most likely due to poor logical understanding, and it is suggested that campaigns before the rollout of such interventions should inform the users that the insecticide are designed to kill malaria causing mosquitoes. However, their presence in the new ITN/LLINs or IRS may irritate other bugs already present in the households and are unable to kill the untargeted burgs due to the low dose or indication of the chemicals [34]. As reported elsewhere, promoting LLINs usage must be accompanied by measures to control bedbugs and other insects of public health importance to avoid problems among communities that can cause poor acceptability and use of these key malaria interventions.

Research has shown that rumours about health interventions tend to decrease over time; they start at the beginning of the campaign and disappear as the campaign intensifies and the community realizing the benefits which tend to outweigh the perceived disadvantages [32]. Rumours around malaria control tools (especially insecticides used in IRS and ITN) with perceived association with erectile dysfunction or impotence, are not new [32, 35]. Such misconceptions can be addressed through strategic health communication, to counter them and show that the LLINs are not toxic, but are rather safe to sleep under [36]. What needs to be looked at here is perhaps why these rumours are sustained after decades of LLINs roll out in different parts of the country. Communities are keen in observing what is happening in their localities; hence claims should not be ignored or overlooked.

Climatic conditions, especially changing levels of temperatures and seasons, have been implicated in influencing the use of LLINs [35–39]. In this study, participants perceived less or no mosquitoes in cooler seasons while warm seasons were thought to have more mosquitoes, and this affects the use of LLINs. Interestingly, in a study of malaria in two regions of Tanzania, participants associated comfort with LLINs use reporting that LLINs provide warmth during cooler seasons, which authors termed non-malaria benefits [35]. Two studies conducted in western [36] and central Kenya [40], reported more or less similar results, where LLINs users in the central parts were more likely to cover under-fives during cooler weather, and their counterparts from Western Kenya valued the LLINs warmth at night but felt uncomfortable during the seasons with high temperatures.

In Zanzibar, where malaria shows a declining trend, LLINs users perceived higher density of mosquitoes during the rainy season, perceiving the burden of malaria and associated nuisance, to also be high [39]. These results communicate two main opposing messages, that LLINs are beneficial regardless of the original intended use and non-malaria benefits, and secondly, they can be a hindrance to personal comfort. These messages may suggest that some of the bed net users are subconsciously trading health benefits to comfort, but with the ongoing malaria trends elsewhere, it is anticipated that health benefits will surpass subsidiary benefits.

The debate on the linkages between poverty and malaria including LLINs and use of other interventions is somewhat complex. Findings from this study suggest that affordability was a key factor to net ownership and use. Studies have shown that having one LLINs in the household contributes to declining mosquito density [41]. Therefore, to sustain the gains made in malaria control, distribution of free (or subsidized) LLINs in areas with persisting malaria transmission should be an ongoing strategy, as recommended by WHO and according to the national guidelines. Special attention should be given to populations at high risk such as pregnant women and children under five years of age, and strong supervision during distribution to ensure each household receives sufficient number of nets to reach the desired ratio of a net per every two people [7, 9, 10, 42]. However, LLINs effectiveness depends on proper usage; hence the community’s inability to afford proper bedding as shown in this study is a serious concern.

Due to reported changes of mosquito biting behaviour [43], malaria control strategies targeting outdoor biting mosquitoes are important to attain and sustain low malaria transmission and achieve elimination [44]. Although larviciding and IRS are perceived as a secondary malaria control strategy in Tanzania [45], it was interesting to have them reported by study participants. Targeting breeding sites, both natural and man-made has been shown to contribute to the decrease in malaria burden elsewhere [34]. The ongoing campaigns in the country [46] need to be enhanced and sustained in areas where malaria burden remains high [47]. The findings showed that in the regions where these strategies are implemented, respondents appreciated their potential to control malaria vectors and exhibited willingness to utilize them.

The concerns raised were attributed to unfaithfulness in the handling and storage of IRS and/or larviciding chemicals by some implementers. Furthermore, the managerial challenges such as a lack of close supervision during mixing and storage of IRS/larviciding chemicals reported in this study serve as a wakeup call for program implementers to employ monitoring mechanisms to ensure the chemicals for larviciding and IRS are effectively used.

Malaria health seeking behaviour and care experience is known to have a significant impact on malaria case management and their potential for reducing severity of the disease [48]. Effective management of malaria includes prompt treatment seeking, followed by correct diagnosis using rapid diagnostic tests, effective treatment and adherence to treatment regimen. Self-medication, visiting traditional healers and the use of local herbs as reported in this study are not effective means of managing uncomplicated and/or complicated/severe malaria as such practices fail to clear parasitaemia and contribute to onward progression of the disease, other undesirable outcomes, and potential transmission of parasite that are resistant to anti-malarial drugs. These practices have been reported as common in Tanzanian communities due to perceived loss of effectiveness of anti-malarials, poor patient provider relationship, inability to pay for treatment services, shortages of drugs, long waiting time and distance to health facilities [22, 49]. With increasing availability of health facilities in rural areas through the strategies to strengthen primary healthcare in Tanzania, provision of better services needs to be strengthened. They should focus on strengthening and adherence to malaria diagnosis guidelines, enforcing strict provision of anti-malarial drugs preceded with an authorised prescription and avoiding over-the counter purchase of artemisinin-based combinations and other anti-malarials. Health system functioning issues and challenges which were reported in this study also need to be urgently addressed. They included distance to health facilities, anti-malarial stock outs, health financing and cost related to seeking care which led to dissatisfaction with the CHF services, and absenteeism of health workers which compounds the already understaffed health facilities in resource-limited settings [50, 51]

### Study limitations

This study despite its usefulness in informing the malaria control approaches has some limitations. First, participants’ perceptions and opinions were not time-bound, hence may not directly reflect the situation of the health system at the time of the survey. That is also compounded with recall bias, variations in intensity of the individual experiences whose details were beyond the scope of this study. Second, when assessing the use of LLINs, it was not always easy to distinguish if a participant was talking about treated or untreated bed nets. Lastly, this study was done in regions where malaria persistence has been observed but no control regions were involved for comparisons.

## Conclusion

The findings showed that study participants had good knowledge of malaria, including its cause, transmission, symptoms and control measures. Misconceptions related to the use of bed nets and IRS, climatic conditions, socio-economic status, behavioural issues, use of local herbs for treating malaria, self-medication, visiting traditional healers and poor patient adherence to treatment regimens were the factors reported to be potentially associated with persistence of malaria in north-western and southern regions of Tanzania. Health system related factors such as inadequate number of health workers, distance to facilities, health financing, cost associated with accessing care and treatment, and limited use of CHF were also reported and need to be addressed. Future analysis should triangulate these findings when assessing the factors behind malaria persistence in these regions. Relevant policies and targeted malaria interventions for reducing and finally eliminating the disease should be put in place in order to reach the ultimate goal of malaria elimination in Tanzania by 2030.

## Data Availability

The data used in this study are available from the corresponding author on a reasonable request.

## Abbreviations

ACT: Artemisinin-based combination therapy
ALu: Artemether–lumefantrine
BCC: Behaviour Change Communication
CHF: Community health fund
DMO: District Medical Officer
FGD: Focus group discussion
IDI: In-depth interview
IPTp: Intermittent preventive treatment in pregnancy
IRS: Indoor residual spraying
ITNs: Insecticide-treated nets
KAP: Knowledge, attitude and practice
LLINs: Long-lasting insecticidal nets
DMFP: District Malaria Focal Person
MRCC: Medical Research Coordinating Committee
NIMR: National Institute for Medical Research
NMCP: National Malaria Control Programme
SMPS: School Malaria Parasitological Survey
TDHS: Tanzania Demographic and Health Survey
TDHS-MIS: Tanzania Demographic and Health Survey and Malaria Indicator survey
THMIS: Tanzania HIV/AIDS and Malaria Indicator Survey
